# The complete chloroplast genome sequence of *Garcinia anomala* (Clusiaceae) from Yunnan Province, China

**DOI:** 10.1080/23802359.2021.1934175

**Published:** 2021-06-15

**Authors:** Biying Yue, Jipu Shi

**Affiliations:** a Xishuangbanna Tropical Botanical Garden, Chinese Academy of Sciences, Mengla, China; b University of Chinese Academy of Sciences, Beijing, China

**Keywords:** Chloroplast genome, *Garcinia anomala*, phylogenetic analysis

## Abstract

*Garcinia anomala* Planch. & Triana is an evergreen tree classified to the genus *Garcinia* in the family Clusiaceae. Here we report and characterize the complete chloroplast genome sequence of *G. anomala* and its phylogenetic relationship was investigated. The chloroplast genome is 156,774 bp in length and has a typical quadripartite chromosomal structure. The genome is divided into a pair of inverted repeat regions (IR) of 27,053 bp, one small single-copy (SSC) region of 17,082 bp and a large single copy (LSC) region of 85,586 bp. The overall GC content is 36.1%. A total of 130 functional genes were annotated, including 85 protein-coding, 37 tRNA and 8 rRNA genes. The phylogenetic analysis of *G. anomala* fully resolved it in a clade with four *Garcinia* taxa within clusioid clade of the Malpighiales.

*Garcinia anomala* (heterotypic synonym *Garcinia bracteata* C. Y. Wu ex Y. H. Li 1981), is an understory tree classified in the genus *Garcinia.* The species is distributed in Yunnan, Guangxi Province in China and north of Indo-China (Planchon and Triana 1860; Wang et al. 2017). In the Dai People culture, its fruit is considered edible and has high economic and medicinal value (Liu et al. 2016). Xanthones, which are known as a unique class of biologically active compounds, are extracted from *G. anomala* and function as antioxidants, antimicrobial, antiviral, cytotoxic, antiinflammation and serve as a broad-spectrum of anti-tumor activities (Niu et al. 2017; Qing et al. 2020). As an important medicinal and horticultural plant, there is a total lack of phylogenetic and genomic data. To better understand its unique chemical components and important pharmacological properties, here the complete chloroplast genome of *G. anomala* is reported.

Fresh leaves of *G. anomala* was collected from the Xishuangbanna Tropical Botanical Garden (XTBG), Chinese Academy of Sciences, Yunnan Province (Location: E 101.2546, N 21.9263, 564 m). The voucher was deposited at the Herbarium of XTBG (http://hitbc.xtbg.ac.cn, Jianwu Li and ljw@xtbg.org.cn) under the voucher number SY6021. Total genomic DNA was extracted using the CTAB method (Doyle and Dickson 1987). The complete chloroplast genome was sequenced following Zhang et al. (Zhang et al. 2016) and their 15 universal primer pairs were used to perform long-range PCR for next-generation sequencing. The contigs were aligned using the publicly available plastid genome of *G. mangostana* (KX822787) and annotated in Geneious 8.1.9.

The plastome of *G. anomala* (MW582313) has a length of 156,774 bp. There are 130 genes in number, including 85 protein-coding, 37 tRNA and 8 rRNA genes. The complete chloroplast genome is comprised of a pair of inverted repeat (IR) regions (27,053 bp) that are separated by a large single copy region (LSC) with a length of 85,586 bp, and a small single copy region (SSC) with a length of 17,082 bp. The total GC content of the chloroplast genome is 36.1%, of which the GC content for the IR regions is 42.1%, the LSC (33.5%) and SSC (30.3%) regions.

In order to confirm the evolutionary relationship of *G. anomala*, a maximum likelihood (ML) phylogenetic tree was inferred based on 78 plastid encoded protein genes, of which 9 species from the order Malpighiales, including 4 species of genus *Garcinia*, and 2 species of Rhizophora that served as the outgroups (Figure 1). The 12 sequences were aligned were aligned using the default settings with the MAFFT version 7 (Katoh et al. 2019). The maximum likelihood phylogenetic analyses were performed based on GTR + F + I + G4 model in the iqtree version 1.6.7, with 1,000 bootstrap replicates (Lam-Tung et al. 2015). The analysis shows that *G. anomala* is fully resolved in a clade containing four other *Garcinia* taxa, and these findings are consistent with former studies (Jin et al. 2020), the clusioid clade of the Malpighiales, which is comprised of five families, is also supported. The complete chloroplast genome sequence of *G. anomala* will be useful for development of further phylogenetic analysis and secondary metabolite studies.

**Figure 1.**
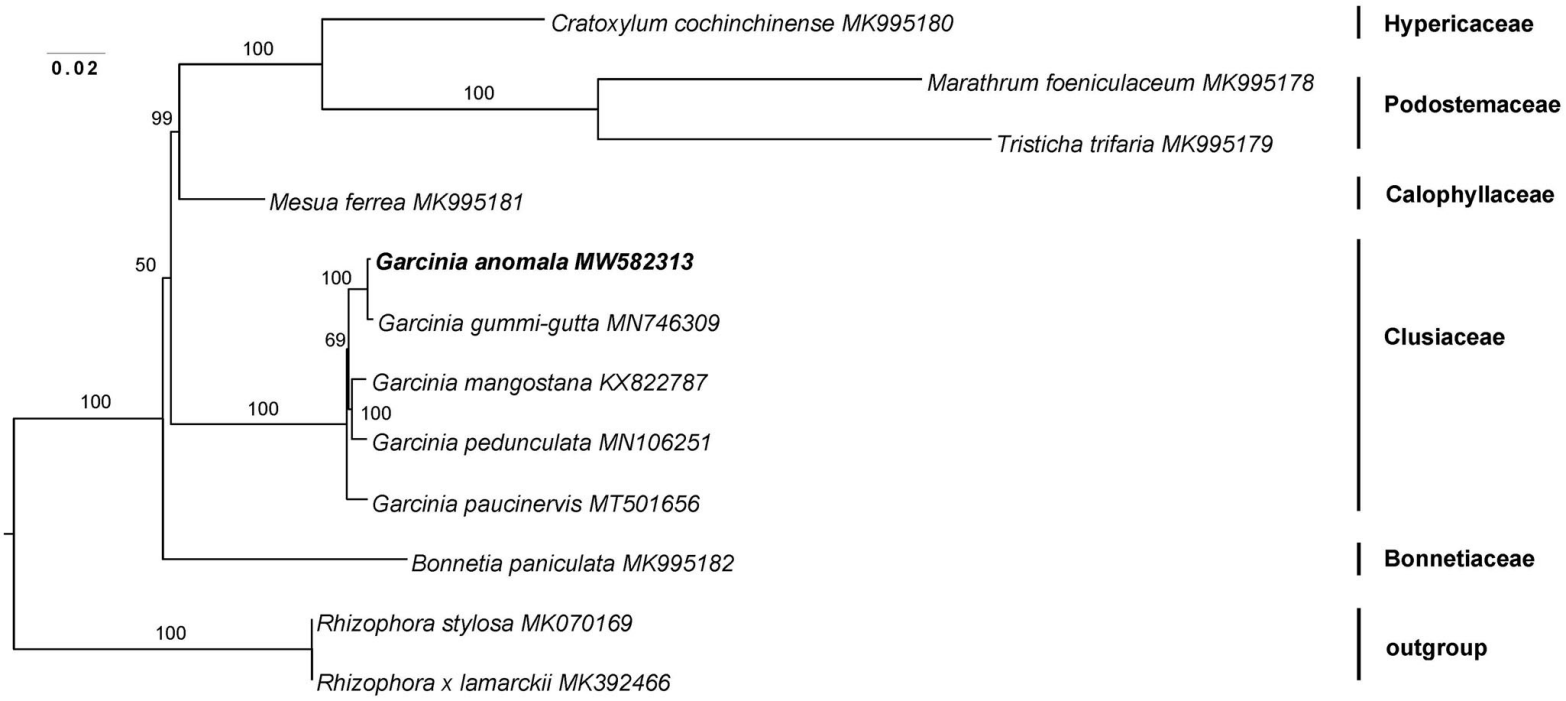
The ML phylogenetic tree for *G. anomala* versus 11 other chloroplast genomes of species in the Malpighiales. Numbers above/below the branches represent ML bootstrap values based on 1,000 replicates.

## Data Availability

The genome sequence data that support the findings of this study are openly available in GenBank of NCBI at [https://www.ncbi.nlm.nih.gov] (https://www.ncbi.nlm.nih.gov/) under the accession no. MW582313. The associated BioProject, SRA, and BioSample numbers are PRJNA726157, SRX10710705, and SAMN18921341 respectively.
